# Efficacy of and tolerance to mild induced hypothermia after out-of-hospital cardiac arrest using an endovascular cooling system

**DOI:** 10.1186/cc5956

**Published:** 2007-06-28

**Authors:** Nicolas Pichon, Jean Bernard Amiel, Bruno François, Anthony Dugard, Caroline Etchecopar, Philippe Vignon

**Affiliations:** 1 Intensive Care Unit, Dupuytren University Hospital, 2 Avenue Martin Luther King, 87000, Limoges, France; 2 Department of Cardiology, Dupuytren University Hospital, 2 Avenue Martin Luther King, 87000, Limoges, France

## Abstract

**Introduction:**

We evaluated the efficacy of and tolerance to mild therapeutic hypothermia achieved using an endovascular cooling system, and its ability to reach and maintain a target temperature of 33°C after cardiac arrest.

**Methods:**

This study was conducted in the medical-surgical intensive care unit of an urban university hospital. Forty patients admitted to the intensive care unit following out-of-hospital cardiac arrest underwent mild induced hypothermia (MIH). Core temperature was monitored continuously for five days using a Foley catheter equipped with a temperature sensor. Any equipment malfunction was noted and all adverse events attributable to MIH were recorded. Neurological status was evaluated daily using the Pittsburgh Cerebral Performance Category (CPC). We also recorded the mechanism of cardiac arrest, the Simplified Acute Physiologic Score II on admission, standard biological variables, and the estimated time of anoxia. Nosocomial infections during and after MIH until day 28 were recorded.

**Results:**

Six patients (15%) died during hypothermia. Among the 34 patients who completed the period of MIH, hypothermia was steadily maintained in 31 patients (91%). Post-rewarming 'rebound hyperthermia', defined as a temperature of 38.5°C or greater, was observed in 25 patients (74%) during the first 24 hours after cessation of MIH. Infectious complications were observed in 18 patients (45%), but no patient developed severe sepsis or septic shock. The biological changes that occurred during MIH manifested principally as hypokalaemia (< 3.5 mmol/l; in 75% of patients).

**Conclusion:**

The intravascular cooling system is effective, safe and allows a target temperature to be reached fairly rapidly and steadily over a period of 36 hours.

## Introduction

Mild induced hypothermia (MIH) was recently shown to improve neurological outcomes in patients who had sustained post-resuscitation encephalopathy secondary to cardiac arrest [1-3]. Accordingly, this procedure has been recommended as part of the standard of care for out-of-hospital cardiac arrest [4]. Nevertheless, the optimal technique for achieving MIH and its benefit/risk ratio in the target population remain controversial [5]. Conventional techniques for effecting therapeutic hypothermia are cumbersome and time consuming, or they do not allow precise control of body temperature [6]. Accordingly, we assessed the efficacy of and tolerance to a recently available endovascular cooling system in patients who were successfully resuscitated following out-of-hospital cardiac arrest.

## Materials and methods

All studied patients underwent MIH as part of their initial management using the CoolGard™ Thermal Regulation System (Alsius Corporation, Irvine, CA 92618, USA) connected to a balloon-equipped endovascular Icy™ catheter (Alsius Corp.) (single perfusion line with cooled normal saline) designed to be inserted in the inferior vena cava via the femoral vein. The decision regarding whether perform MIH was left to the discretion of the attending physician. Exclusion criteria were age above 85 years, a Glasgow Coma Scale score above 7 on admission, an in-hospital cardiac arrest, an estimated time of anoxia in excess of 40 min, and time from initiation of advanced cardiac life support (ACLS) to recovery of spontaneous circulation greater than 60 min. Because this observational study did not alter the standard of care in resuscitated patients after cardiac arrest in our institution, no informed consent was required.

### Cooling system

**Figure 1:**
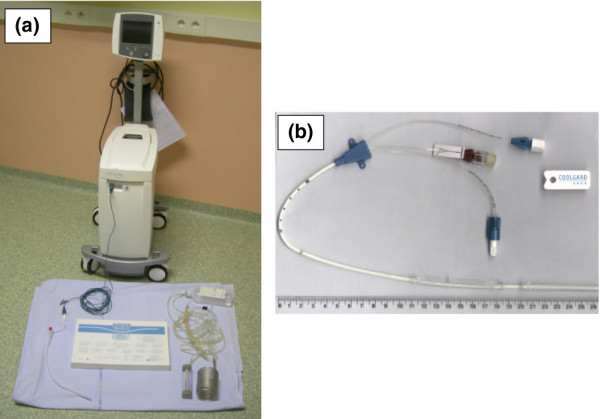
Equipment used. **(a) **Inserted CoolGard™ Thermal Regulation System with patient's temperature monitoring, and **(b) **balloon-equipped Icy™ femoral catheter used for endovascular cooling.

The femoral catheter was 35 cm long with three inserted cylindrical balloons, which were filled with serum saline and connected to a bedside refrigerator designed to reach and maintain a target temperature set by the operator (Figure 1). Normal saline temperature in the cooling system was automatically adjusted according to the patient's core temperature, which was monitored using a temperature-sensing thermistor bladder catheter, and the target temperature and the desired rate of cooling (ranging from 0.1°C/hour to 0.7°C/hour) set by the operator.

### Mild induced hypothermia

No prehospital hypothermia was induced and the MIH procedure was initiated as soon as possible after the patient was admitted to the emergency room. The target temperature was usually 33°C, and a maximal cooling rate was typically chosen. Once the target temperature was obtained, MIH was usually maintained for 36 hours. The core temperature was subsequently increased at a rate of 0.3°C/hour using the cooling system.

### Patient management

ACLS was performed according to standard guidelines [7]. Efforts were made to use the 'Utstein' style for reviewing, reporting and conducting research on post-resuscutation care [8,9]. All patients were intubated endotracheally in the field and were mechanically ventilated by the prehospital medical team. Central venous lines, including the femoral catheter, were inserted by the intensivist, who was on call in the emergency room on admission. No radiological assessment was performed to check that the tip of the catheter was positioned within the inferior vena cava. Patients received routine acute clinical care, including monitoring of vital signs. Whether emergency coronarography should be performed was left to the discretion of the cardiologist and intensivist in charge of the patient. When angioplasty was performed, routine anti-aggregant treatment associated with unfractioned heparin was administered. MIH was interrupted only during patient transportation to the coronarography room, and it was resumed during the revascularization procedure. Patients were sedated with a continuous midazolam infusion, and pancuronium bromide was administered when necessary to avoid shivering during MIH.

### End-points

The primary end-point was the ability of the endovascular cooling system to achive a preset target temperature and to maintain a steady MIH of 33°C for 36 hours, which was empirically defined as variations in core temperature of less than 0.4°C. Secondary objectives were to describe expected side effects that had previously been attributed in the literature to MIH or to the cooling system; to assess spontaneous core temperature variations after cessation of MIH; and to evaluate patient neurological outcome, as assessed using the Pittsburgh Cerebral Performance Category (CPC) on day 28 [1,10,11].

### Data collection

Core temperature was monitored continuously for five days and recorded every two hours during the first 12 hours, every six hours for the next three days, and twice a day for the remaining days. A Foley catheter equipped with a temperature sensor CURITY^® ^(Degania Silicone Ltd, Degania Bet, Israel) was used to monitor core temperature (range of measured temperature: 0°C to 50°C, with an accuracy of ± 0.1°C between 25°C and 45°C). The time of initiation of ACLS was defined as T_0_. Any equipment malfunction was noted and all adverse events attributable to MIH were recorded during hypothermia and until core temperature reached 37°C after rewarming.

In each patient, neurological status was evaluated daily until hospital discharge or death, or on day 28, whichever occurred first, by phone call to the patient or their family. This evaluation was performed by one independent physician, who had not been involved in patient care, using the CPC, which is based on the Glasgow Outcome Performance categories [8,12]. A CPC score of 1 or 2 is consistent with a favorable neurological outcome, whereas a CPC score of 3, 4, or 5 reflects a poor neurological outcome or death.

We also recorded the mechanism of cardiac arrest (ventricular fibrillation or tachycardia versus pulseless rhythms), Simplified Acute Physiologic Score II on admission, standard biological variables, and the estimated time of cerebral anoxia. The time of onset of cardiac arrest was only recorded in cases of witnessed cardiac arrest, and the estimated duration of cerebral anoxia was defined as the interval from collapse (presumed time of cardiac arrest) to first resuscitation attempt by emergency medical services. Biological variables were recorded every 12 hours for three days and once daily after. Because variations in body temperature have an important impact on the results of blood gas monitoring, algorithms were used to correct arterial blood gases for MIH [13]. No particular infection control strategy was applied during MIH. Guidelines on nosocomial infections were applied to define pneumonia, urinary tract infection, or catheter-related septicemia [14]. Systematic ultrasound of the femoral veins was performed after catheter removal to exclude potential development of deep vein thrombosis.

### Ethics committee

This study was approved by the Association des Réanimateurs du Centre-Ouest ethics committee, which waived the need for informed consent.

### Statistical analysis

Continuous variables are expressed as mean ± standard deviation. Categorical variables are reported as number (percentage). Mann-Whitney *U *test was used to compare continuous variables and χ^2 ^test (or Fischer's exact test when necessary) was used to compare categorical variables between subsets of patients with favorable and poor neurological outcomes. *P *< 0.05 was considered statistically significant.

## Results

Over a two-year period, 81 patients were admitted to the intensive care unit of our institution for management of resuscitated cardiac arrest. Among them, 10 patients (12%) sustained in-hospital cardiac arrest and were excluded. Among the 71 patients who sustained an out-of-hospital cardiac arrest, 31 patients (44%) had at least one exclusion criterion. The remaining 40 patients underwent MIH and constituted the study population.

Cardiac arrest was considered to be of cardiac origin in 31 patients (78%) and was deemed hypoxic in the nine remaining patients (three drownings, four hangings and two penetrative injuries). Among the 31 patients, the initial rhythm was ventricular fibrillation in 14, asystole in 23 and pulseless electric activity in the remaining three. Emergency angioplasty was performed in nine patients (23%), after initiation of MIH. The patients' characteristics are summarized in Table 1.

**Table 1:** Characteristics of the study population at baseline and outcome

Characteristic	All patients (*n *= 40)	Outcome^a^		
		Good (*n *= 13)	Poor (*n *= 27)	*P *value

Mean age (years)	58 ± 14	53 ± 17 (15 to 76)	61 ± 12 (30 to 81)	0.12
SAPS II score	61 ± 19	49 ± 18 (13 to 75)	66 ± 17 (41 to 125)	0.01
Men (*n*)	31/40	8/13 (62%)	23/27 (85%)	0.23
Ventricular fibrillation	14	8 (57%)	6 (43%)	
Asystole	23	4 (17%)	19 (83%)	
Other pulseless electric activity	3	1 (33%)	2 (67%)	
Temperature on admission to the emergency department (°C)	36 ± 1	36.1 ± 1.1 (33.8 to 38)	35.9 ± 1.1 (33.6 to 37.7)	0.86
Glasgow Coma Scale score	4 ± 1	4 ± 2 (3 to 7)	3 ± 1 (3 to 7)	0.20
Glucose on admission (mmol/l)	13.6 ± 5	14.6 ± 4.9 (9 to 24.4)	13.1 ± 5.1 (2.6 to 22.2)	0.55
Lactates on admission (mmol/l)	9.8 ± 6.4	8.4 ± 5.8 (2.3 to 19.1)	10.5 ± 6.6 (2.4 to 24.8)	0.20
Arterial pH on admission	7.25 ± 0.18	7.25 ± 0.15 (6.93 to 7.45)	7.25 ± 0.19 (6.84 to 7.54)	0.55
Estimated time of anoxia (min)	11 ± 9	7 ± 4 (0 to 14)	13 ± 9 (1 to 40)	0.03
Time from initiation of ACLS to ROSC (min)	16 ± 10	21 ± 12 (1 to 45)	14 ± 8 (1 to 35)	0.06
Time from ACLS to initiation of MIH (min)	98 ± 54	103 ± 50 (45 to 190)	96 ± 57 (55 to 300)	0.51
Time from initiation of MIH to achieving goal temperature (min)	187 ± 119	198 ± 88 (30 to 360)	181 ± 132 (30 to 600)	0.25

Values are expressed as mean ± standard deviation. ^a^Good outcome corresponded to CPC scores of 1 or 2; poor outcome corresponded to CPC scores of 3 to 5. ACLS, advanced cardiac life support; CA, cardiac arrest; MIH, mild induced hypothermia; ROSC, return of spontaneous circulation; SAPS, Simplified Acute Physiology Score.

Mean interval between ACLS and initiation of MIH was 98 ± 54 min (range 45 to 300 min), and the target core temperature was reached after a mean interval of 296 ± 148 min (range 110 to 805 min) after cardiac arrest. Catheters were inserted successfully in all cases. The target temperature of 33°C was achieved after a mean period of 187 ± 119 min (range 30 to 600 min) after initiation of MIH (Table 1). In one patient, the intervals between cardiac arrest and initiation of MIH and between initiation of MIH and stabilization at the target temperature were 205 min and 600 min, respectively. This patient exhibited a relatively high body temperature on admission (37°7C), partly related to refractory myoclonus, and did not receive paralysis treatment. Once the target temperature was achieved, active cooling was performed for 37 ± 6 hours (range 20 to 48 hours).

Six patients (15%) died (four from cardiogenic shock and two from cerebral death) during MIH. Cardiac dysrythmia was observed in 11 patients (28%) during hypothermia, but this appeared to be unrelated to MIH. Dysrythmia consisted of ventricular fibrillation in two patients (who underwent defibrillation) and atrial fibrillation in the remaining nine patients (who received intravenous amiodarone, associated with correction of hypokalaemia in five of them).

**Figure 2:**
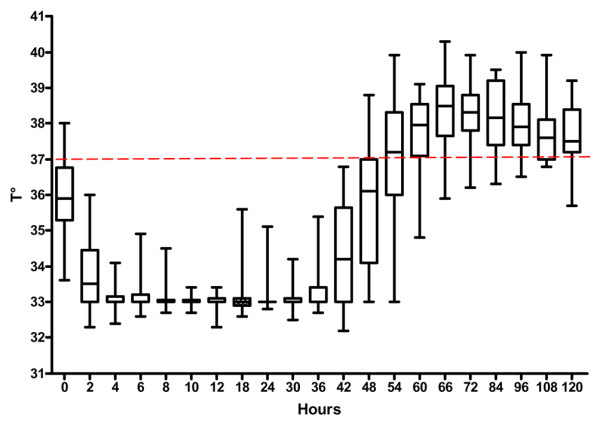
Evolution of core temperature during MIH and rewarming in the study population. MIH, mild induced hypothermia.

Pancuronium bromide was administered to 19 patients (48%) to avoid excessive shivering during MIH. Among the 34 patients who completed the 36-hour period of MIH, hypothermia was maintained steadily in 31 patients (91%), with a core temperature maintained between 32.6°C and 33.4°C (Figure 2). In the three remaining patients, maximal temperature variations recorded around the target temperature were 1°C, 1.5°C and 2.6°C.

Once progressive rewarming was initiated, normothermia was achieved after a mean of 808 ± 365 min (range 420 to 1,800 min), close to the expected 800 min necessary at a warming rate of 0.3°C/hour. Post-rewarming 'rebound hyperthermia', defined as temperature of 38.5°C or greater, was observed in 25 patients (74%) during the first 24 hours that followed cessation of MIH (Figure 2). Catheters were withdrawn during the 24 hours following the end of MIH. None of the catheters were found to be colonized. No deep venous (particularly femoral) thrombosis was diagnosed both clinically and with routine ultrasound at the time of catheter removal.

Only few complications attributable to MIH were observed (Table 2). Haemorrhagic complications consisted mainly of nonserious bleeding related to the venous catheter insertion, which did not require blood transfusion. A single case of traumatic false aneurysm formation secondary to accidental puncture of a femoral artery was encountered (Table 2). Infectious complications were observed in 18 patients (45%), but no patient developed severe sepsis or septic shock. Five patients developed a nosocomial bacteraemia (*Staphylococcus aureus *in four cases), five patients (13%) were diagnosed with an early-onset nosocomial pneumonia (occurring within 72 hours after tracheal intubation; Table 2), six patients were diagnosed with an early-onset nosocomial bronchitis, and two patients were diagnosed with an urinary tract infection. During the study period, rates of infection in patients who did not undergo MIH in our intensive care unit were 12% for nosocomial pneumonia, 14% for bronchitis, 7% for nosocomial urinary tract infections and 2% for nosocomial bacteraemia. There was no apparent relationship between these documented bacterial infections during MIH and post rewarming 'rebound hyperthermia'.

**Table 2:** Complications observed during MIH and 5 days after rewarming and patients' outcome

Adverse effects	Complications (*n *[%])	Outcome^a^	
		Good (*n *= 13)	Poor (*n *= 27)

Bleeding of any severity	3 (8%)	1 (8%)	2 (8%)
Need for packed RBC transfusions	4 (10%)	2 (16%)	2 (8%)
Complication on catheter site^b^	3 (8%)	1 (8%)	2 (8%)
Hypokalaemia (< 3.5 mmol/l)	30 (75%)	10 (77%)	20 (74%)
Nosocomial infection (MIH/non-MIH)^c^			
Septicemia	5 (13%)/2%^c^	2 (16%)	3 (11%)
Urinary tract infection	2 (5%)/7%^c^	2 (16%)	0
Pneumonia	5 (13%)/12%^c^	2 (16%)	3 (11%)
Bronchitis	6 (15%)/14%^c^	2 (16%)	4 (15%)

^a^Good outcome corresponded to CPC scores of 1 or 2; poor outcome corresponded to CPC scores of 3 to 5. ^b^Included are haematoma, local haemorrhage and false aneurysm formation. ^c^This percentage is the rate of infection in patients who did not undergo MIH in our intensive care unit during the study period. MIH, mild induced hypothermia; RBC, red blood cells.

Biological pancreatitis and seizures were not observed. When compared with baseline values obtained on admission, MIH presumably resulted in mild biological changes, with the exception of relevant hypokalaemia; 28% of patients had a potassium level below 3.5 mmol/l on admission, whereas this proportion reached 77% after 24 hours of MIH. Consequently, the mean potassium level significantly decreased after 24 hours of MIH compared with baseline (3.2 ± 0.6 mmol/l [range 2.2 to 4.6 mmol/l] versus 4.1 ± 0.8 mmol/l [range 2.8 to 6.6 mmol/l]; *P *< 0.0001). During the initial course of hypothermia, potassium was monitored closely and maintained within the normal range.

Among the 27 patients (67%) with a poor outcome, 24 patients died during their hospitalization (six patients during MIH and 18 patients after a decision to withdraw acute care) and three patients had moderate or severe neuromotor disability at hospital discharge. The remaining 13 out of the 40 patients had a favorable neurological outcome, with a CPC score of either 1 (*n *= 8) or 2 (*n *= 5) on day 28. Overall, 57% of patients who sustained a ventricular fibrillation, 17% of patients with asystole and 33% of patients with other pulseless electric activity had a favorable outcome. The estimated time of anoxia was shorter in the subset of patients with favorable outcome (7 ± 4 min versus 13 ± 9 min; *P *= 0.03). The time between initiation of ACLS and return of spontaneous circulation and the time between ACLS and initiation of MIH were similar between groups (Table 1). Similarly, the period required to achieve the target core temperature was similar between study groups (Table 1). MIH-attributed complications were equally distributed between patient groups (Table 2).

## Discussion

MIH is increasingly being used to provide protection for the brain against post-ischaemic injury [15]. In various clinical circumstances, such as resuscitation following cardiac arrest, MIH may improve neurologic outcome when it is initiated quickly enough [1-4]. Clinical studies evaluating both the efficacy of and tolerance to recently available endovascular cooling systems are scarce [16].

The present study showed that the CoolGard™ Thermal Regulation System, connected to an Icy™ catheter inserted in the femoral vein, allowed the target temperature of 33°C to be attainted after a mean of 187 min (Table 1). This intravascular cooling system appeared to induce hypothermia faster than external devices, because a similar target temperature was obtained after means of 480, 301 and 287 min using external cooling techniques (cold air mattress, ice packs and cooling blankets, respectively) [1,10,17]. External cooling systems usually allow body temperature to be decreased at a rate of 0.3 to 0.5°C/hour [18], whereas the endovascular device used in our patients induced hypothermia at a mean rate of 1.1 ± 0.4°C/hour without use of additional external device. Paralytic drug therapy, frequently used in our patients, presumably accelerated the cooling rate by avoiding excessive shivering. Using the same endovascular cooling device, Georgiadis and coworkers [19] recently reported a mean cooling rate of 1.4 ± 0.6°C/hour. In keeping with previous reports, the present study suggests that intravascular cooling systems allow induction of moderate hypothermia more rapidly than various external techniques.

Because the protective effects of MIH appear to be greater when it is initiated early [20], investigators have recently proposed that ice cold intravenous fluid be administered to reduce the time needed to reach the target temperature [3,6,21]. In 22 patients who sustained an out-of-hospital cardiac arrest, Bernard and coworkers [3] lowered body temperature by 1.6°C over 25 min by rapidly infusing 30 ml/kg of crystalloid at 4°C, without noticeable adverse effect. Similarly, Polderman and coworkers [6] recently reported mean decreases in core temperature of 2.3°C and 4.0°C over 30 min and 60 min, respectively, in 134 patients undergoing MIH. Although more rapid than the endovascular device evaluated in the present study, rapid infusion of refrigerated saline does not allow one to maintain induced hypothermia steadily at the predefined target temperature. Accordingly, this approach appears to be a promising additional means to induce hypothermia rapidly, but it should be combined with another system once the target temperature has been reached [6].

In the present study, the target temperature of 33°C was reached in all patients and maintained steadily over a mean of 36 hours in all but three patients (91%). In contrast, external cooling systems such as cooling blankets, ice packs, cold lavage, or cooling helmet failed to achieve the target temperature in a substantial proportion of patients in whom MIH was indicated [1,10,17]. In a landmark clinical study, Holzer and coworkers [1] reported that external cooling using packs of ice allowed attainment of the target temperature of 33°C in only 30% of patients hospitalized after cardiac arrest. Furthermore, with regard to temperature variation with time, MIH was fairly stable over time (37 ± 6 hours) in our patients (Figure 2), whereas core temperature was found to be less stable in studies using external cooling techniques [1,10,17].

Although slow rewarming is widely advocated, because rapid increase in body temperature after MIH may offset its potential beneficial effects on brain injury [11], no precise recommendation is currently available regarding the optimal rewarming technique [4]. In 74% of our patients, we observed an increase in body temperature to 38.5°C or greater during the first 24 hours that followed progressive rewarming (Figure 2). This finding is in keeping with that reported by McIntyre and coworkers [18], who observed 'rebound hyperthermia' resulting from rapid rewarming after MIH induced to manage severe head trauma with associated brain oedema. Importantly, this rebound hyperthermia was associated with an increase in intracranial pressure. Brain oedema secondary to postanoxic injury after a cardiac arrest may potentially be worsened by the hyperthermia that was noted in a substantial proportion of our patients after slow rewarming. This phenomenon frequently observed after rewarming has not yet been clearly explained, and may be secondary to sustained cytokine release related to ischaemia/reperfusion cerebral injuries. In the present study, prolonged use of the cooling system (with a target temperature of 37°C) appears to be an effective approach to avoiding post-rewarming rebound hyperthermia in patients who underwent MIH after cardiac arrest. Nevertheless, efficacy of and tolerance to such prolonged use of this external cooling system require further study.

The present study showed that early-onset nosocomial infections were the most frequent complication observed over seven days after the initiation of MIH (Table 2). Comparison of rates of nosocomial infections between patients who underwent MIH and the remaining patients hospitalized in our intensive care unit over the same period revealed an increased rate of bacteraemia only (13% versus 2%). A significantly higher rate of nosocomial pneumonia during therapeutic hypothermia was reported only in one case-control study [22], but this finding was not confirmed by our study (13% versus 12%). Hypokalaemia was observed in the majority of our patients, but this was not associated with relevant arrhythmia (Table 2). A nonsignificant increase in white blood cells was observed during MIH (*P *= 0.02), whereas haemoglobin and platelet count remained unchanged. In a recent meta-analysis [23], only one clinical study identified a nonsignificant increase in haemorrhagic complications and sepsis in patients undergoing MIH when compared with normothermic patients [1]. In our study, no complication related to insertion of the 35 cm femoral catheter was noted. Minor local complications such as bleeding at the puncture site have previously been reported in fewer than 10% of cases [19,24].

The present observational study has several limitations. In the absence of randomization, the potential benefits of MIH in terms of neurological outcome cannot be evaluated in our study population. Similarly, reported complications in our patients cannot clearly be attributed to MIH. In addition, MIH was maintained for a mean of 37 ± 6 hours, whereas the International Liaison Committee on Resuscitation currently recommends a period of 24 hours [4]. Nevertheless, the Committee emphasizes the fact that the optimum duration of hypothermia remains to be determined [4] and the potential benefits of applying MIH for longer than 24 hours require further investigation [25]. Some of our patients who sustained a hypoxic cardiac arrest with asystole also underwent MIH, but they had a poor neurological outcome. According to the literature and the International Liaison Committee on Resuscitation recommendations, patients suffering out-of-hospital cardiac arrest secondary to asystole should not undergo MIH [26]. Although MIH is currently recommended in witnessed cardiac arrest secondary to ventricular fibrillation or tachycardia [4], it may be performed in other categories of patients regardless of initial rhythm, provided that the cardiac arrest is witnessed and the prognosis is not hopeless because of associated comorbidities [11]. Finally, physicians in charge of resuscitated patients who have suffered cardiac arrest recently reported fairly low rates of application of MIH [27]. Incorporation of current resuscitation guidelines for MIH and future research aimed at improving cooling techniques may improve physicians' awareness and utilization of therapeutic hypothermia after out-of-hospital cardiac arrest [27-29].

## Conclusion

The present study demonstrated that the CoolGard™ system combined with the Icy™ venous catheter is efficient at inducing MIH and well tolerated. This intravascular cooling system allowed attainment of a target temperature of 33°C fairly rapidly and maintenance of MIH steadily over a period of 36 hours in all patients. This system was also consistently effective at progressive rewarming. Ease of use, efficacy and tolerance to this cooling system justify further studies to evaluate modalities for inducing MIH in various clinical settings, aiming to optimize the protective effect against post-ischaemic and traumatic brain injury. The efficacy and safety of prolonged use of this endovascular cooling system to avoid post-rewarming rebound hyperthermia remain to be investigated, as well as the potential relation between MIH and early-onset nosocomial infections.

## Key messages

• The Alsius CoolGard™ intravascular cooling system combined with the Icy™ venous catheter is a safe, efficient and well tolerated method for inducing MIH.

• This intravascular cooling system appears to be more efficient than conventional external cooling methods in tightly regulating body temperature (induction and stability of mild therapeutic hypothermia, as well as its reversal).

• The safety and efficiency of prolonged use (> 24 hours) of the present cooling system remain to be determined by further studies.

• The potential relation between mild therapeutic hypothermia and early-onset nosocomial infection remains to be investigated, especially regarding the development of bacteraemia.

• Incorporation of therapeutic hypothermia into resuscitation guidelines may increase physicians' awareness and yield improvements in the use of cooling systems in resuscitated patients after cardiac arrest.

## Abbreviations

ACLS = advanced cardiac life support; CPC = Pittsburgh Cerebral Performance Category; MIH = mild induced hypothermia.

## Competing interests

The authors declare that they have no competing interests.

## Authors' contributions

NP and JBA were responsible for the conception and design of the study, acquisition and interpretation of data, and writing of the manuscript. BF, AD and CE acquired data. PV was responsible for reviewing and writing of the manuscript.
